# Antioxidant Defenses in the Kidneys and Heart of the Freshwater Fish *Astyanax lacustris* Subjected to High (31°C) and Low (15°C) Temperatures

**DOI:** 10.1002/cbf.70133

**Published:** 2025-10-28

**Authors:** Ana Paula Nascimento Corrêa, Luiz Neves Neto, Maria Rosa Dmengeon Pedreiro de Souza, Niumaique Gonçalves da Silva, Jonathan Ratko, Ananda Karla Alves Neundorf, Ieda Cristina Schleger, Tatiana Herrerias, Lucélia Donatti

**Affiliations:** ^1^ Laboratory of Adaptive Biology, Department of Cell Biology Federal University of Paraná Curitiba Paraná Brazil; ^2^ Federal University of Paraná—Postgraduate Program on Ecology and Conservation Curitiba Paraná Brazil; ^3^ Federal University of Paraná—Postgraduate Program on Cellular and Molecular Biology Curitiba Paraná Brazil; ^4^ Health Sciences Center Federal University of Santa Catarina Florianópolis Santa Catarina Brazil

**Keywords:** biomarkers, stress responses, subtropical fish, thermal stress, yellow‐tailed lambari

## Abstract

Aquatic ecosystems have their abiotic and biotic factors constantly altered by various factors. Among them, water temperature is an abiotic factor that can significantly affect fish physiology, increasing energy demand, which can impact homeostasis and survival. Endocrine and metabolic changes and enzymatic modulation are referred to as stress responses, which can lead to oxidative stress, generating negative physiological effects when temperature limits are exceeded. Oxidative stress biomarkers used in combination can highlight the effects of a stressful condition. Here, we seek to understand how the species *Astyanax lacustris*, which is native to Brazil and has ecological and economic importance, as well as remarkable research potential, responds to changes in water temperature. Thus, we evaluated the effects of high (31°C ± 1°C) and low (15°C ± 1°C) thermal stress on the antioxidant defense system in the heart and kidneys of *A. lacustris*. Specimens were collected from artificial lakes in União da Vitória (PR) and exposed to different temperatures for periods of 2, 6, 12, 24, 48, 72, or 96 h, with a control group mantained at 23°C ± 1°C. The results indicated that in the heart exposed to 31°C, there was modulation in the biomarkers superoxide dismutase (SOD), glutathione peroxidase (GPx), and glutathione (GSH), while at 15°C only GPx activity was altered. In the kidneys of fish exposed to 31°C, there was a change in the activity of the biomarkers catalase (CAT), glutathione‐S‐transferase (GST), and lipid peroxidation (LPO), while at 15°C there was modulation of the glutathione reductase (GR) biomarker and changes in the levels of reactive oxygen species (ROS). Responses to heat stress were organ‐specific, influenced by temperature and exposure time. Principal component analysis (PCA) indicated an association of glutathione‐dependent biomarkers at high temperatures in the kidneys, while responses in the heart were similar across temperatures. Overall, *A. lacustris* exhibited distinct antioxidant responses in different tissues under thermal stress, with kidney response being more sensitive to heat, while cardiac responses were less variable across treatments.

## Summary

- Heat stress profoundly affects intracellular organelles and disrupts cellular metabolism, leading to altered biochemical homeostasis.
- Elevated temperatures promote oxidative stress through increased production of reactive oxygen species (ROS), impacting mitochondrial function, endoplasmic reticulum stability, and redox‐sensitive signaling pathways.
- These changes require the rapid activation of antioxidant defenses to preserve cellular integrity. In contrast, cold exposure induces a more gradual metabolic response, reflecting differential regulation of enzymatic activity and organelle adaptation.
- Our results highlight the dynamic role of organelles in sensing and responding to thermal fluctuations and emphasize how heat stress modulates the biochemical regulation of cellular function, energy balance, and oxidative signaling in ectothermic organisms.

## 1 Introduction

Changes in environmental temperature significantly affect fish physiology, influencing aspects such as food consumption, survival, growth, reproduction, behavior, and distribution [1], and these effects vary depending on the acclimatization capacity of the species to environmental changes [2]. Cases of mass mortality in fish during extreme winter and summer conditions, which affect physiological homeostasis and osmotic and ionic regulation, have been reported (C [3]). In addition, climate change caused by global warming simultaneously impacts the biotic and abiotic factors of the environment, generating stress, especially for aquatic species [4, 5, 6, 7].

Fish are constantly exposed to environments with varying temperatures [8], and because they are ectothermic, they can adjust to these fluctuations. Higher temperatures tend to increase the respiratory rate and may increase the production of reactive oxygen species (ROS), whereas lower temperatures decrease cellular metabolism (Z [9]). ROS are highly reactive molecules that, when produced in excess, can damage essential cellular structures such as proteins, lipids, and DNA. These damages can be detected by biomarkers including lipid peroxidation (MDA), protein carbonylation (PCO), and oxidative DNA lesions (8‐oxo‐dG). ROS are normally produced by metabolism, but ROS production can increase under adverse environmental conditions, leading to cellular oxidative stress [10, 11]. These highly reactive molecules can cause damage to proteins, lipids and DNA [12, 13, 14]. An increase in ROS generation may trigger antioxidant defense adjustment responses [15, 16]; Z [9, 17].

Cells possess a set of enzymes with antioxidant activities that can be used as biomarkers of the stress response, including superoxide dismutase (SOD), catalase (CAT), glutathione‐s‐transferase (GST), and glutathione peroxidase (GPx) [15, 18, 19]. The responses of the antioxidant defense system to thermal stress are specific to the organ, species, temperature, and duration of exposure [20].

*Astyanax lacustris* is a fish found in Brazilian lakes, streams, and rivers [21]. This species has a wide geographic distribution in South America [22] and has been used as an experimental model in studies of thermal stress. *A. lacustris* tolerates a relatively broad temperature range with an optimal thermal window for physiological comfort and normal metabolic function estimated between 24°C and 28°C [23]. Several responses related to antioxidant defenses occur in organs and tissues exposed to thermal stress in *A. lacustris*. In the liver, increases in the enzymatic activity of CAT and GST and the concentration of reduced glutathione (GSH) have been observed after 72 h at 31°C, indicating that the antioxidant defense system was able to respond to a possible increase in ROS formation caused by high‐temperature thermal stress [24]. In the muscle of *A. lacustris*, an increase in the activity of the enzyme glutathione reductase (GR) and in the level of protein carbonylation (PCO) at 15°C has been reported, indicating the occurrence of oxidative stress, whereas at 31°C, GR activity sharply increased after 24 h of exposure [25]. In the brain of *A. lacustris* at 31°C, GPx, glucose‐6‐phosphate dehydrogenase (G6PDH), and GSH were found to be the most responsive enzymes [23]. In addition, in *A. lacustris*, the gills were most responsive to thermal stress in relation to the antioxidant defense system [20]. This finding was corroborated by other studies in which different organs, including the gills, of *Psalidodon bifasciatus*, a species that is phylogenetically close to *A. lacustris*, were analyzed [26].

Temperature directly affects the metabolic rate of fish; higher temperatures increase metabolism and can lead to greater production of nitrogenous waste, generating greater pressure on the kidneys to excrete this waste. In addition, the kidney is a central stress response organ in vertebrates, as it is part of the hypothalamus‒pituitary‒interrenal (HPI) axis, which is responsible for the secretion of cortisol released in response to stress in teleost fish [27]; Z [9]. In the heart, thermal stress is capable of affecting cardiac pacemaking [28], and it is believed that the heart, in addition to being responsible for meeting the increased demand for oxygen, is among the organs most sensitive to temperature elevation [29] owing to the high mitochondrial density and high oxygen consumption, primary sources of ROS [30]. However, the effects of thermal stress on the antioxidant defense system of the kidneys and heart are not yet known. The temperatures of 15°C and 31°C were chosen because they represent the natural extremes observed in the Iguaçu River Basin, while 23°C was selected as the control condition, as it is close to the thermal comfort range of *Astyanax lacustris* (22°C–28°C) [31, 32]. We hypothesize that thermal stress induces distinct antioxidant responses in the kidney and heart of *A. lacustris*.

Thus, the objective of this study was to evaluate the ability of the heart and mid‐posterior kidneys of *A. lacustris* to adjust to high (31°C) and low (15°C) temperatures in relation to the antioxidant defense system, thus helping elucidate the effects of climate change on aquatic organisms.

## 2 Materials and Methods

An environmental license for animal collection was obtained from the Chico Mendes Institute for Biodiversity Conservation (SISBIO/ICMBio), under number 63551‐1, and an animal experimentation license was obtained from the Committee on Ethics in the Use of Animals of the Biological Sciences Sector of Federal University of Paraná (CEUA‐BIO/UFPR), under number 1228 of 10/30/2018.

### 2.1 Collection and Acclimatation of *A. lacustris*

The fish were collected from the Iguaçu River Basin, which presents average temperatures of 15.5°C in winter and 28.8°C in summer [33], with an annual average of 20°C [31]. As collections occurred in more than one event after the last collection, the animals were maintained in artificial lakes until reaching the required sample size (*n* = 330), for approximately 60 days at an average temperature of 23°C. This temperature was chosen based on literature data that indicate the thermal comfort range for *A. lacustris* [32, 34]. The artificial lakes are located at the Center for Research and Extension in Aquaculture Ildo Zago, União ′da Vitória, Paraná, Brazil (26°13′12.15″S; 51°7′51.07″W).

*A. lacustris* specimens were collected with fishing nets composed of multifilament fabric with 12 mm mesh between the nodes of the artificial lakes, transferred to 830 L tanks, and acclimatized for 3 days under controlled conditions to recover from capture stress [20, 24, 35]. The water was constantly aerated, with a continuous flow of 4 L/min, and a natural photoperiod of 13.5 h light and 10.5 h dark was maintained, considering the literature, and the region's sunshine and the month in which the experiments were carried out [36, 37]. In addition, the temperature was controlled at 23°C ± 1°C. Abiotic measures such as dissolved O_2_ (8.44 + 2.17 mg/L) (mean ± SE), ammonia (NH_3_) (0.009 ± 0.004 mg/L), pH (7.4 ± 0.31), nitrate (NO_3_–) (0.00 mg/L), nitrite (NO_2_–) (0.00 mg/L), and the absence of residual chlorine in the water were strictly controlled once per day.

The tanks were static and were cleaned once per day by suction, with approximately 50% of the water being replaced daily [34] by spring water, with a continuous flow of 4 L/minute.

The fish were fed once daily with an average of 1% body weight of commercial fish feed (Supra Aqua Line), which has a protein content of 42% [34, 38]. The first day of acclimatization was the first feeding day for all groups, with feeding occurring simultaneously in the control and experimental groups.

### 2.2 Thermal Shock Experimental Design

After acclimatization, the fish were randomly distributed and transferred directly to 50‐L aquariums with the water maintained at 15°C ± 1°C or 31°C ± 1°C [24, 34], representing low‐ and high‐temperature thermal stress, respectively. Two control groups were maintained at 23°C ± 1°C, simultaneously with their respective experimental groups. At these temperatures, the experimental fishes and their respective controls (23°C ± 1°C) were kept for periods of 2, 6, 12, 24, 48, 72, and 96 h, with a total of 10 individuals per temperature and time. Two different aquariums with five fish per aquarium were used to ensure biological duplicates. In total, 280 specimens of *A. lacustris* of both sexes, with an average length of 6.08 ± 0.84 cm and an average weight of 8.84 ± 3.2 g. The maximum density per aquarium was 1.8 g of fish per liter of water [38, 39]. Dissolved oxygen levels were measured every 24 h, and the mean rates in the experimental aquariums were 8.16 mg/L at 31°C and 8.62 mg/L at 15°C. In the controls (23°C), the levels were 9.25 mg/L and 7.72 mg/L, respectively. The ammonia and pH values in the control and experimental aquariums were similar to those during acclimatization.

The water exchange conditions used in the experiments and the respective controls beyond the first 24 h were identical to those used for acclimation. The water temperature of the aquariums was strictly controlled by thermostats (Aqua One, VigoAr and Atman, with a power of 100 W). The fish kept at 15°C were placed in aquariums inside a horizontal refrigerator (Consul/530 L) with a digital temperature controller (TC‐900E POWER/07) and kept with the lid open to maintain the photoperiod.

The food offered to the control and experimental fish was identical to that used for acclimatization, and the last food was provided between 22 and 24 h before fish euthanasia, thus ensuring that they were in a fasting state at the time of euthanasia. This approach was adopted to minimize any potential interference of the powered state in the measurements of enzymatic activity (Diana [20, 40, 41]). Food intake data were not evaluated in this study because of the biomarkers analyzed.

After each experiment, the fish were anesthetized with 20 mg/L benzocaine and euthanized via spinal cord sectioning, followed by immediate dissection, with the mid‐posterior kidneys and heart collected on ice and stored in liquid nitrogen.

### 2.3 Biochemical Analysis of Oxidative Stress Biomarkers

Samples from the mid‐posterior kidneys and heart of *A. lacustris* were weighed, homogenized in 50 mM Tris‐HCl buffer (pH 7.4), and centrifuged at 12,000*g* and 4°C for 20 min, after which the supernatants were aliquoted and stored at −80°C. For the mid‐posterior kidneys, owing to the size of the samples, pools of two individuals were prepared (*n* = 5), whereas for the heart, the samples were analyzed individually (*n* = 10).

The spectrophotometric experiments were performed in triplicate via an Epoch microplate spectrophotometer (BioTek), with a saturating substrate concentration and ideal pH and room temperature (22°C) conditions. The activity of G6PDH and PCO and the total ROS concentration were measured only in the mid‐posterior kidneys because of the insufficiency of biological heart samples.

Protein determination was performed according to the method proposed by Bradford [42]. Catalase (CAT, EC 1.11.1.6) activity was evaluated by the degradation of hydrogen peroxide and measured by the decrease in absorbance at 240 nm via a spectrophotometer [43]. The activity of superoxide dismutase (SOD, EC 1.15.1.1) was determined by inhibiting the reduction of nitro blue tetrazolium (NBT) to formazan blue via measurement of the absorbance at 560 nm [44]. The activity of glutathione S‐transferase (GST, EC 2.5.1.18) was measured as GST catalysis of the reaction of 1‐chloro‐2,4‐dinitrobenzene (CDNB) with GSH, forming a thioether, and was measured as the increase in absorbance at 340 nm [45].

The activity of glutathione peroxidase (GPx, EC 1.11.1.9) was evaluated through the reduction of oxidized glutathione (GSSG) catalyzed by GR in the presence of NADPH and was measured by the decrease in absorbance at 340 nm [46]. The activity of glutathione reductase (GR, EC 1.8.1.7) was measured through the oxidation of NADPH and evaluated as the decrease in absorbance at 340 nm [47]. The activity of glucose‐6‐phosphate dehydrogenase (G6PDH, EC 1.1.1.49) was measured by the reduction of oxidized nicotinamide adenine dinucleotide phosphate (NADP^+^) in NADPH, and the increase in absorbance at 340 nm [48] was observed.

The concentration of total ROS was determined through the formation of dichlorofluorescein (DCF), with excitation at 485 nm and emission at 530 nm, as described previously [49].

Lipid peroxidation (LPO) was measured via the quantification of malondialdehyde (MDA) present in the sample at 535 nm [50]. PCO was measured through the formation of dinitrophenyl hydrazones, which were assessed by reading at 360 nm [51]. The concentration of GSH was determined through the reaction of DTNB with the nonprotein thiols present in the sample after protein precipitation. The product of this reaction was detected by reading at 415 nm [52].

### 2.4 Statistical Analysis and Graphical Representation

The data obtained were first transformed via the ordered quantile (ORQ) technique [53]. For this purpose, the bestNormalize package was used [54]. The normality and homoscedasticity of the data were verified via the Shapiro–Wilk and Levene tests, respectively. To verify the differences between the control and treatment groups at each time point and between exposure times, each biomarker was subjected to two‐way analysis of variance (ANOVA) (*p* < 0.05). To identify specific differences, Tukey's post hoc test was performed using the emmeans package [55]. The bar graphs were constructed via the ggplot2 package [56]. The data are presented as the means ± standard errors of the means.

The effects of temperature in the groups exposed to both high‐ and low‐temperature stress was compared with those in the control group and the mobilization of biological functions for acclimatization were observed as increasing mean values of the index of biomarker response (IBR) [2, 23, 57]. For this purpose, the antioxidant defense biomarkers SOD, CAT, GST, GPx, GR, and GSH from the heart and kidneys were integrated into a single category. The calculation was performed via the CALculate IBR interface, developed by the Interdisciplinary Laboratory for Continental Environments of the University of Lorraine, France [58]. This platform uses the calculation proposed by Beliaeff & Burgeot [59] and revisited by Devin et al. [60]. The radar graphics were prepared using the ggradar package [61].

The biological information on sensitivity (Sb) [62] was estimated on the basis of the IBR value of the test (IBR2) and control (IBR1) temperature conditions for each duration of exposure and organ, using the formula Sb = ((IBR2 – IBR1)/IBR1) × 100 and expressing as a percentage (%) for high‐ and low‐temperature exposure. Negative and positive percentages indicate a decrease and increase in the sensitivity of the organs, respectively, to the temperatures studied during the exposure times (2, 6, 12, 24, 48, 72, and 96 h).

Principal component analysis (PCA) was performed with the packages factoextra [63] and FactoMineR [64] to investigate which oxidative stress biomarkers were important descriptors of the temperatures analyzed (15°C and 31°C).

Statistical analyses and graphical representations were produced entirely in R software, version 4.4.1 [65].

## 3 Results

During the experiment, a 3.9% *A. lacustris* death rate was observed, including eight fish in the control and three fish in the treatment groups at 15°C. *A. lacustris* maintained at 15°C presented low swimming activity and little ingestion activity, whereas the fish maintained at 31°C presented greater swimming activity and ate fully and immediately after feeding. The F and *p* values of the data below are presented in Table 1 to Supporting Information S1: 4 in the Supporting Material.

**Table 1:** Values of the sensitivity of kidneys and heart of *Astyanax lacustris* under thermal stress at 31°C and 15°C. The values were calculated from the IBR variation values for each tissue according to the exposure time. Negative values correspond to a decrease in the IBR values and indicate that the tissue did not show significant sensitivity. The values are expressed as percentages.

Organ	Time	31°C	15°C
Kidneys	2 h	−7.07	−51.34
	6 h	97.79	−66.02
	12 h	380.17	−80.63
	24 h	−34.41	20.34
	48 h	33.16	−39.74
	72 h	−63.90	101.92
	96 h	113.86	−66.59
Heart	2 h	353.08	−42.18
	6 h	146.15	108.22
	12 h	−8.74	17.70
	24 h	59.79	25.12
	48 h	208.18	222.81
	72 h	−52.15	−33.10
	96 h	453.10	8.81

### 3.1 Effects of High (31°C) and Low (15°C) Temperature Thermal Shock on the Mid‐Posterior Kidneys of *A. lacustris*

**Figure 1:**
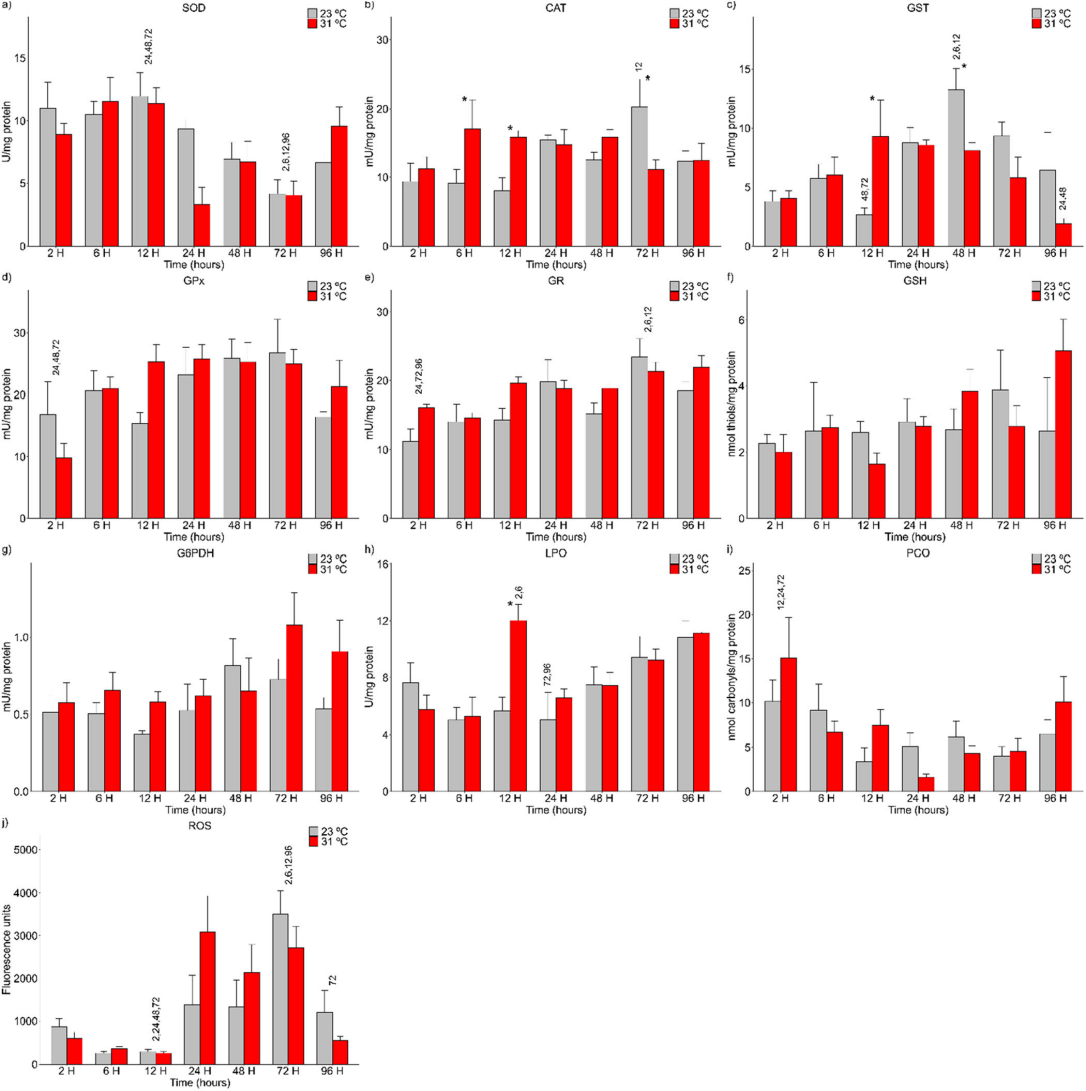
Levels of antioxidant defense biomarkers in the mid‐posterior kidneys of Astyanax lacustris exposed to high‐temperature thermal shock (31°C) and their respective controls (23°C). The asterisks (*) represent significant differences (*p* < 0.05) between the control and experimental groups. The numbers above the bars represent the variation between the different exposure times. The values are presented as the means and standard errors of the means. A—SOD, superoxide dismutase; B—CAT, Catalase; C—GST, Glutathione S‐transferase; D—GPx, Glutathione peroxidase; E—GR, Glutathione reductase; F—G6PDH, Glucose‐6‐phosphate dehydrogenase; G—LPO, Lipid peroxidation; H—PCO, Protein carbonylation; I—GSH, Reduced glutathione; J—ROS, Reactive oxygen species.

In the mid‐posterior kidneys of *A. lacustris* exposed to 31°C, CAT (*p* = 0.010) activity (Figure 1b) increased at 6 and 12 h and decreased at 72 h. The GST (*p* = 0.032) activity (Figure 1c) increased at 12 h, followed by a decrease at 48 h. There was also an increase in LPO (*p* = 0.027) at 12 h (Figure 1h) of exposure. These variations were observed when the control (23°C) and experimental (31°C) groups were compared.

The activities of the biomarkers SOD (Figure 1a), GPx (Figure 1d), GR (Figure 1e), G6PDH (Figure 1g), PCO (Figure 1i), GSH (Figure 1f), and ROS (Figure 1j) in the mid‐posterior kidneys of *A. lacustris* were not significantly different between the control and experimental groups.

**Figure 2:**
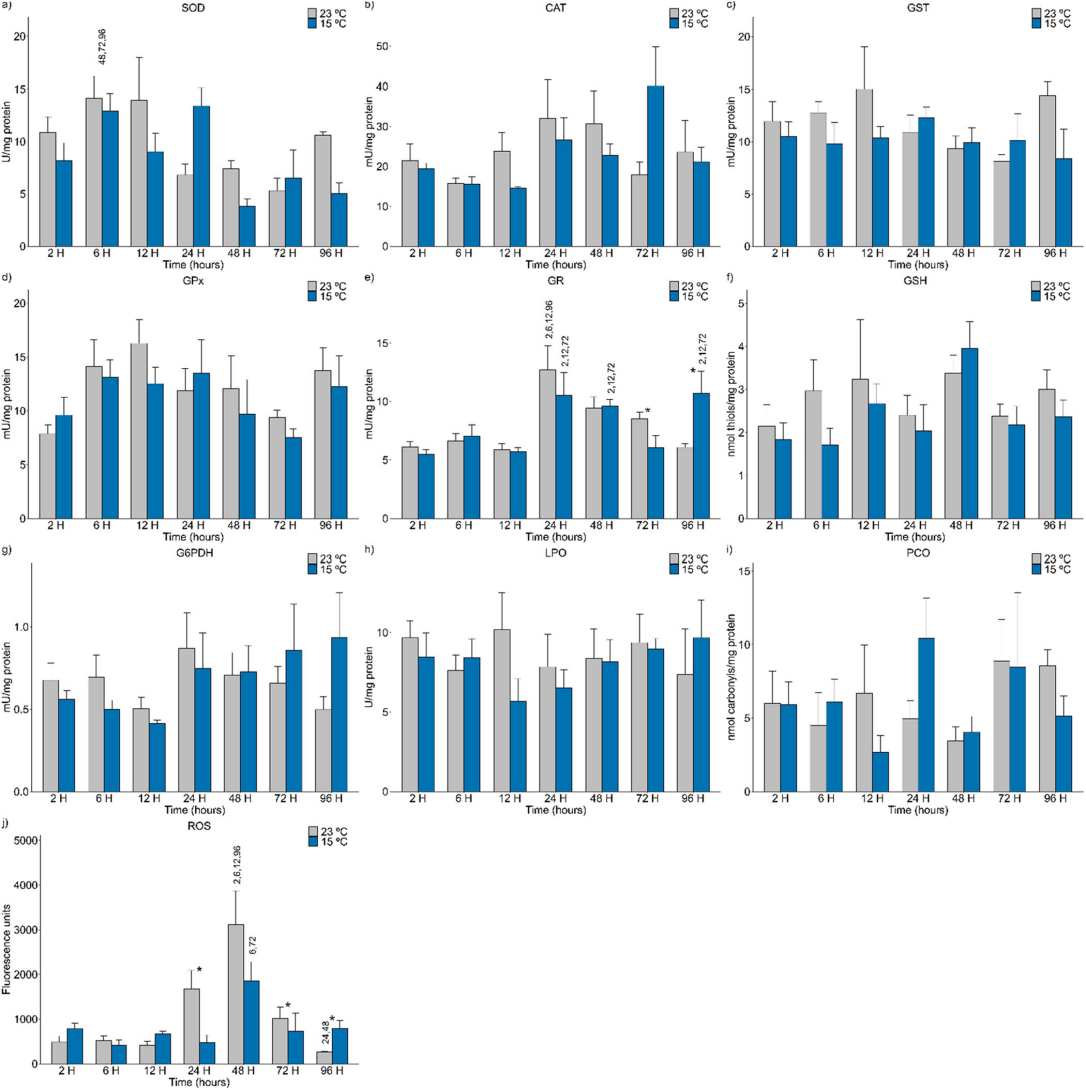
Levels of biomarkers of antioxidant defense in the mid‐posterior kidneys of Astyanax lacustris exposed to low‐temperature thermal shock (15°C) and their respective controls (23°C). The asterisks (*) represent significant differences (*p* < 0.05) between the control and experimental groups. The numbers above the bars represent the variation between the different exposure times. The values are presented as the means and standard errors of the means. A—SOD, superoxide dismutase; B—CAT, Catalase; C—GST, Glutathione S‐transferase; D—GPx, Glutathione peroxidase; E—GR, Glutathione reductase; F—G6PDH, Glucose‐6‐phosphate dehydrogenase; G—LPO, Lipid peroxidation; H—PCO, Protein carbonylation; I—GSH, Reduced glutathione; J—ROS, Reactive oxygen species.

At low temperature (15°C), the GR (*p* = 0.016) biomarker in the mid‐posterior kidneys changed, decreasing at 72 h and subsequently increasing at 96 h of exposure (Figure 2e). The ROS (*p* = 0.001) levels (Figure 2j) decreased at 24 and 72 h, with a subsequent increase at 96 h of exposure to 15°C compared with those of the control (23°C).

The levels of SOD (Figure 2a), CAT (Figure 2b), GST (Figure 2c), GPx (Figure 2d), and G6PDH (Figure 2g) activity and the LPO (Figure 2h), PCO (Figure 2i), and GSH contents (Figure 2f) did not significantly differ between the control (23°C) and experimental (15°C) groups in the mid‐posterior kidneys of *A. lacustris*.

### 3.2 Responses of Antioxidant Defense Biomarkers at the Different Temperatures in the Mid‐Posterior Kidneys of *A. lacustris*

**Figure 3:**
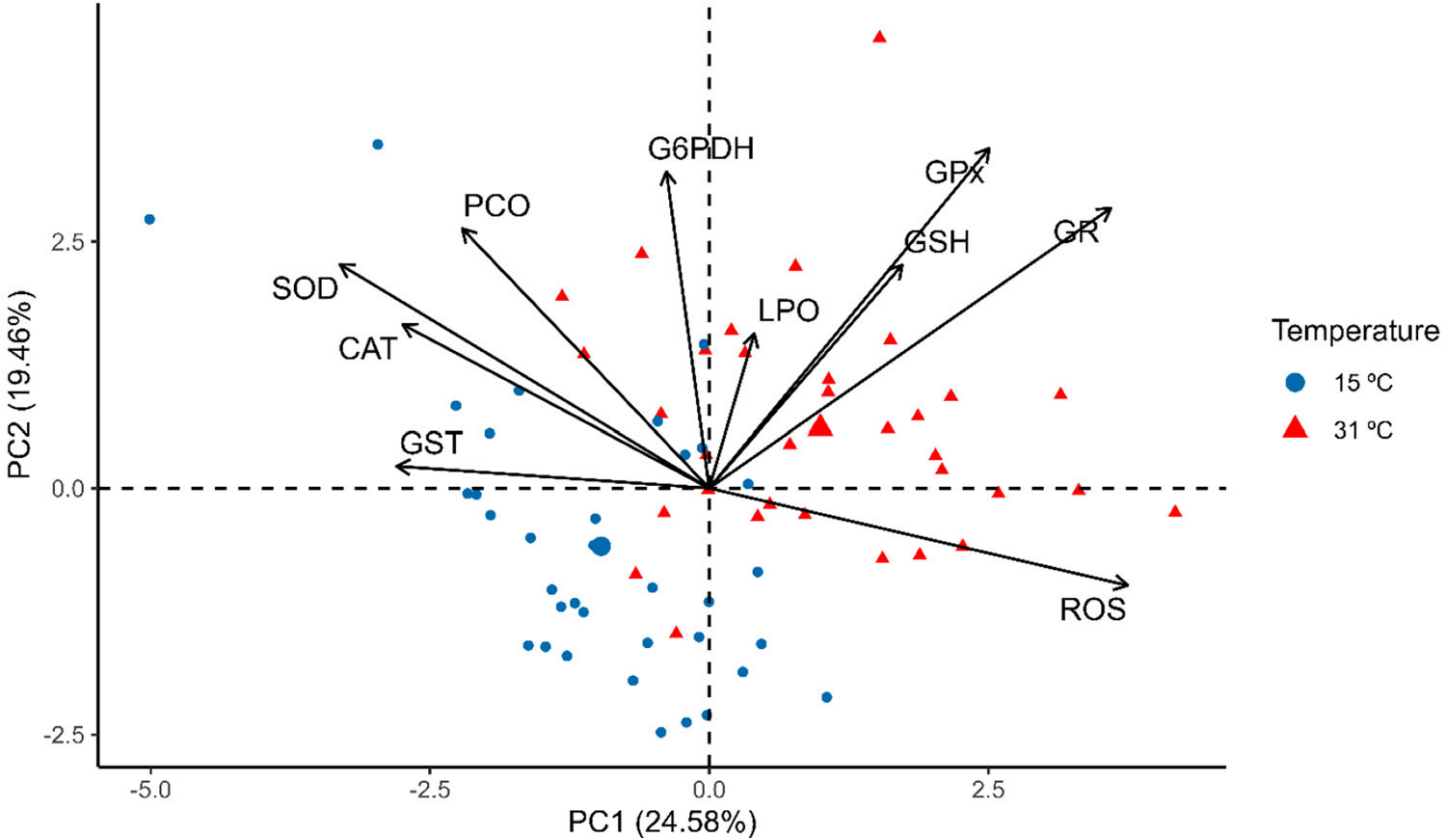
Graph of the principal component analysis (PCA) of biomarkers in the mid‐posterior kidneys of *Astyanax lacustris* exposed to 15°C and 31°C. SOD, Superoxide dismutase; CAT, Catalase; GPx, Glutathione peroxidase; GR, Glutathione reductase; GST, Glutathione s‐transferase; G6PDH, Glucose‐6‐phosphate dehydrogenase; GSH, Reduced glutathione; LPO, Lipoperoxidation; PCO, Protein carbonylation; ROS, Reactive oxygen species.

The variation in kidney antioxidant defense markers at 31°C and 15°C was subjected to PCA: 44.04% of the variation was explained, with 24.58% in the first axis and 19.46% in the second axis. The biplot in Figure 3 indicates that the biomarkers G6PDH, GSH, GR, GPx, LPO, and ROS were correlated with 31°C, while SOD, CAT, GST, and PCO were correlated with 15°C.

### 3.3 Integrated Index of Biomarker Response (IBR) for High (31°C) and Low (15°C) Thermal Shock Temperature in the Mid‐Posterior Kidneys of *A. lacustris*

**Figure 4:**
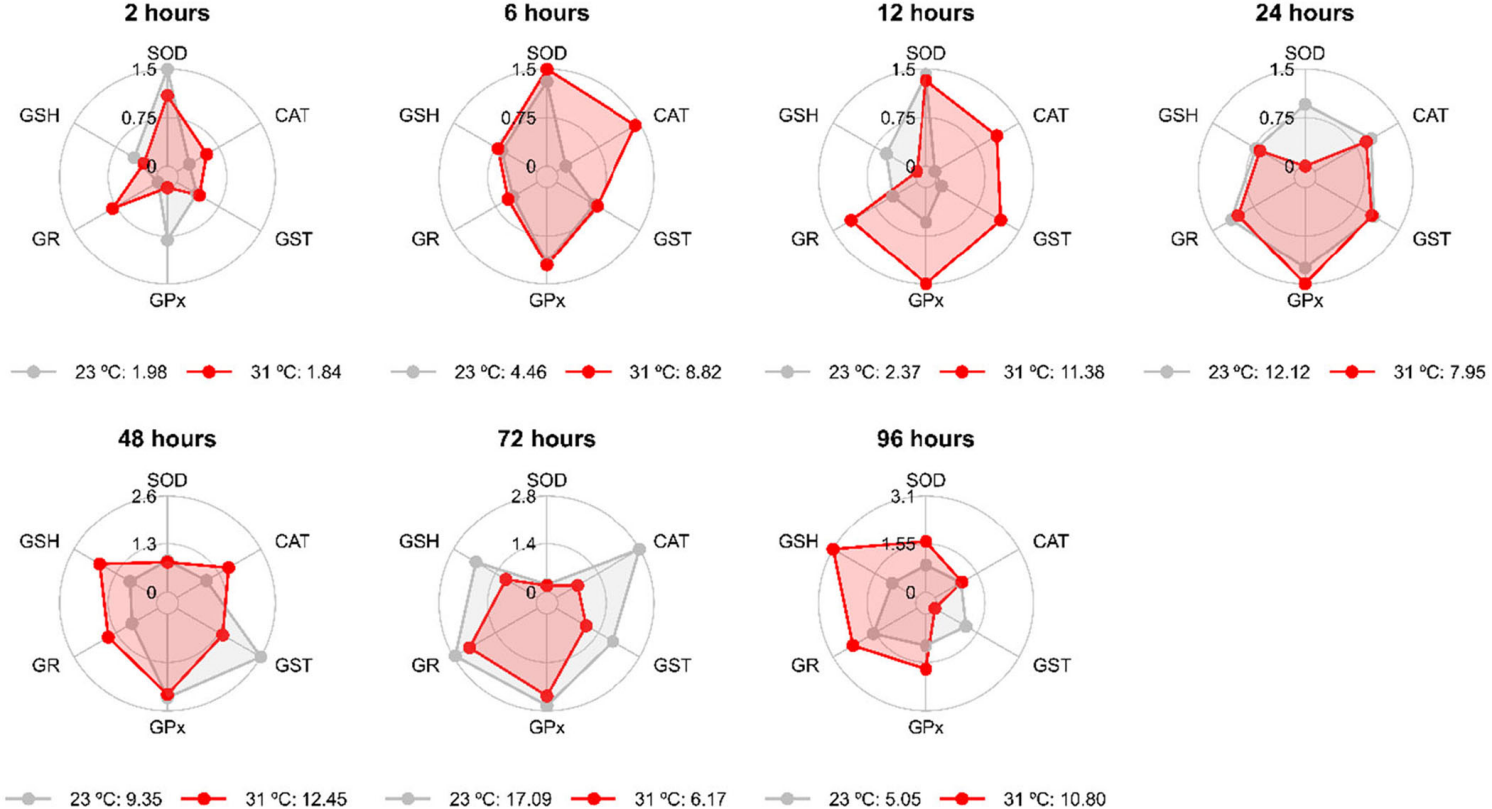
Values of the integrated index of biomarker response (IBR) of the mid‐posterior kidneys of *Astyanax lacustris* exposed to high temperature (31°C) for 2, 6, 12, 24, 48, 72, and 96 h. CAT, catalase; GST, glutathione S‐transferase; GPx, glutathione peroxidase; GR, glutathione reductase; GSH, reduced glutathione; SOD, superoxide dismutase.

**Figure 5:**
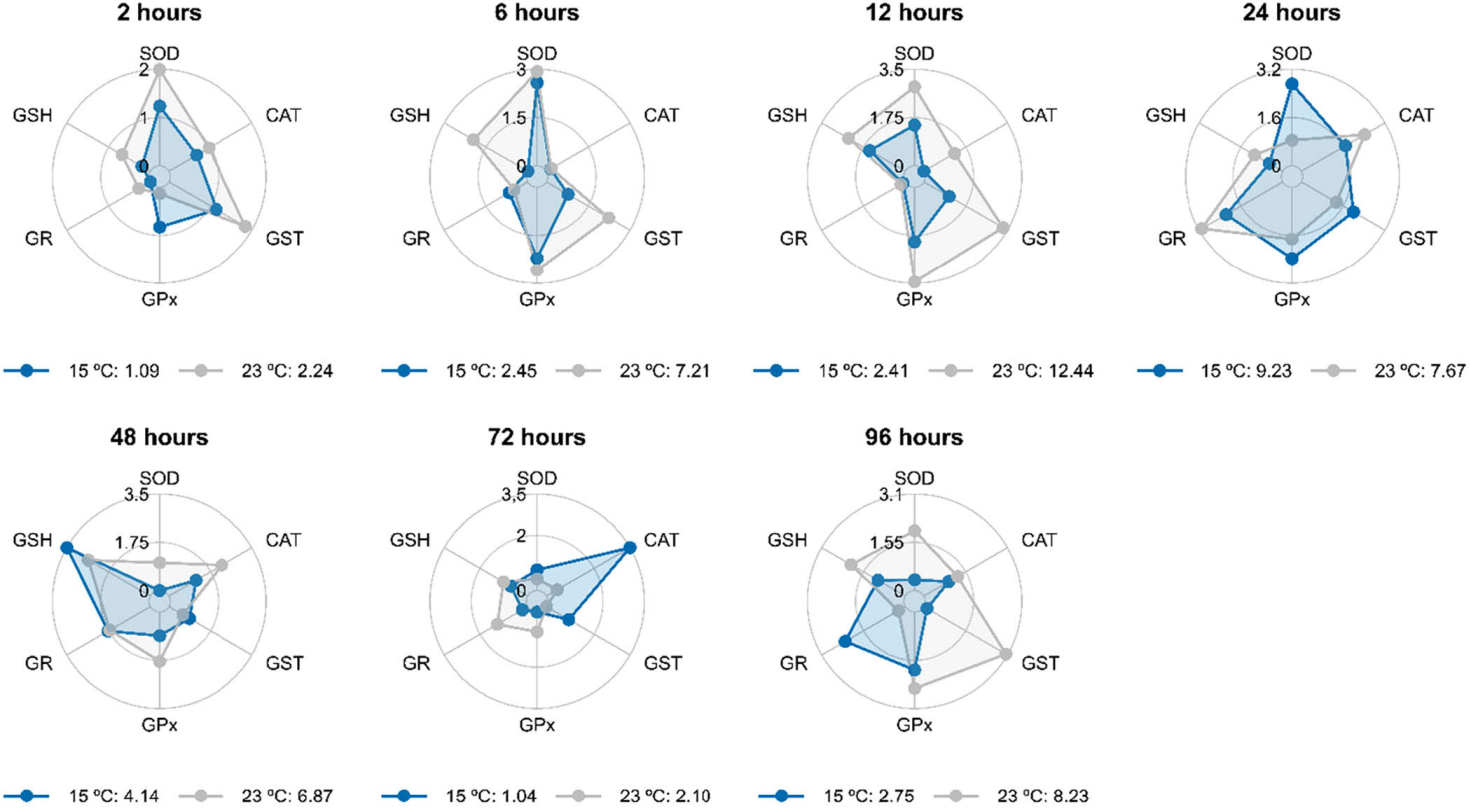
Values of the integrated index of biomarker response (IBR) of the mid‐posterior kidneys of *Astyanax lacustris* exposed to low temperature (15°C) for 2, 6, 12, 24, 48, 72, and 96 h. CAT, catalase; GST, glutathione S‐transferase; GPx, glutathione peroxidase; GR, glutathione reductase; GSH, reduced glutathione; SOD, superoxide dismutase.

The exposure of *A. lacustris* kidneys to 31°C (Figure 4) revealed that the most responsive biomarkers of antioxidant defense for the evaluated times were as follows: GR at 2 h; SOD, CAT, GST, and GPx at 6 h; CAT, GST, GPx, and GR at 12 h; GPx at 24 h; CAT, GR, and GSH at 48 h; and SOD, GPx, GR, and GSH at 96 h (Figure 5). The most responsive biomarkers at 15°C (Figure 5) were GPx at 2 h; SOD, GST, and GPx at 24 h; GSH at 48 h; CAT and GST at 72 h; and GR at 92 h.

### 3.4 Effects of High (31°C) and Low (15°C) Temperature Thermal Shock on the Heart of *A*. *lacustris*

**Figure 6:**
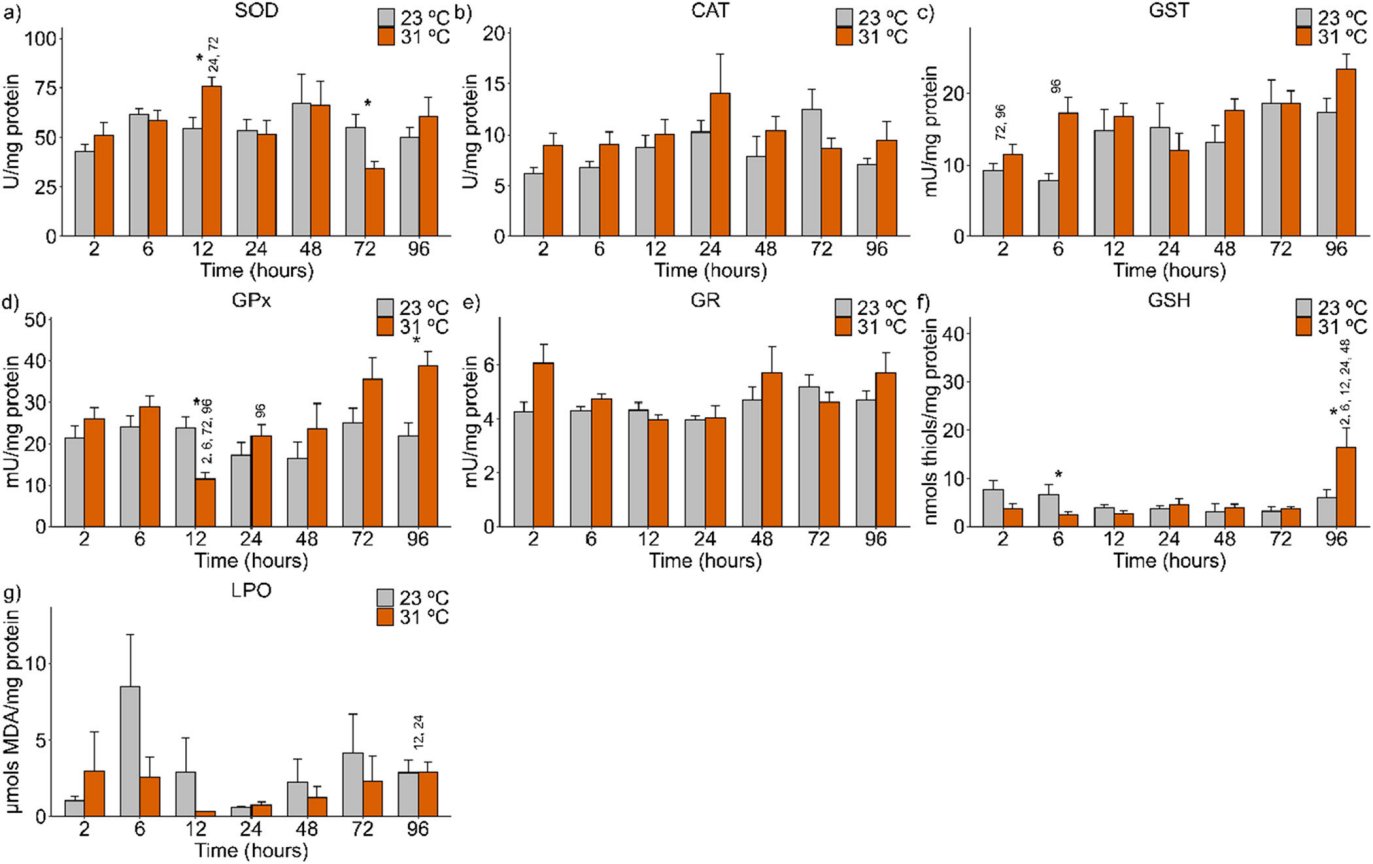
Levels of antioxidant defense biomarkers in the heart of *Astyanax lacustris* exposed to high‐temperature thermal shock (31°C) and their respective controls (23°C). The asterisks (*) represent significant differences (*p* < 0.05) between the control and experimental groups. The numbers above the bars represent the variation between the different exposure times. The values are presented as the means and standard errors of the means. A—SOD, superoxide dismutase; B—CAT, Catalase; C—GST, Glutathione S‐transferase; D—GPx, Glutathione peroxidase; E—GR, Glutathione reductase; F—GSH, Reduced glutathione; G—LPO, Lipid peroxidation.

At 31°C, SOD (*p* = 0.045) activity in the heart of *A. lacustris* increased at 12 h but decreased at 72 h (Figure 6a). The activity of GPx (*p* = 0.002) decreased at 12 h but increased at 96 h (Figure 6d). GSH (*p* = 0.006) contents decreased and then increased at 6 and 96 h, respectively (Figure 6f).

The expression of the markers CAT (Figure 6b), GST (Figure 6c), GR (Figure 6e), and LPO (Figure 6g) in the hearts of *A. lacustris* did not differ at 31°C compared with the respective controls at 23°C.

**Figure 7:**
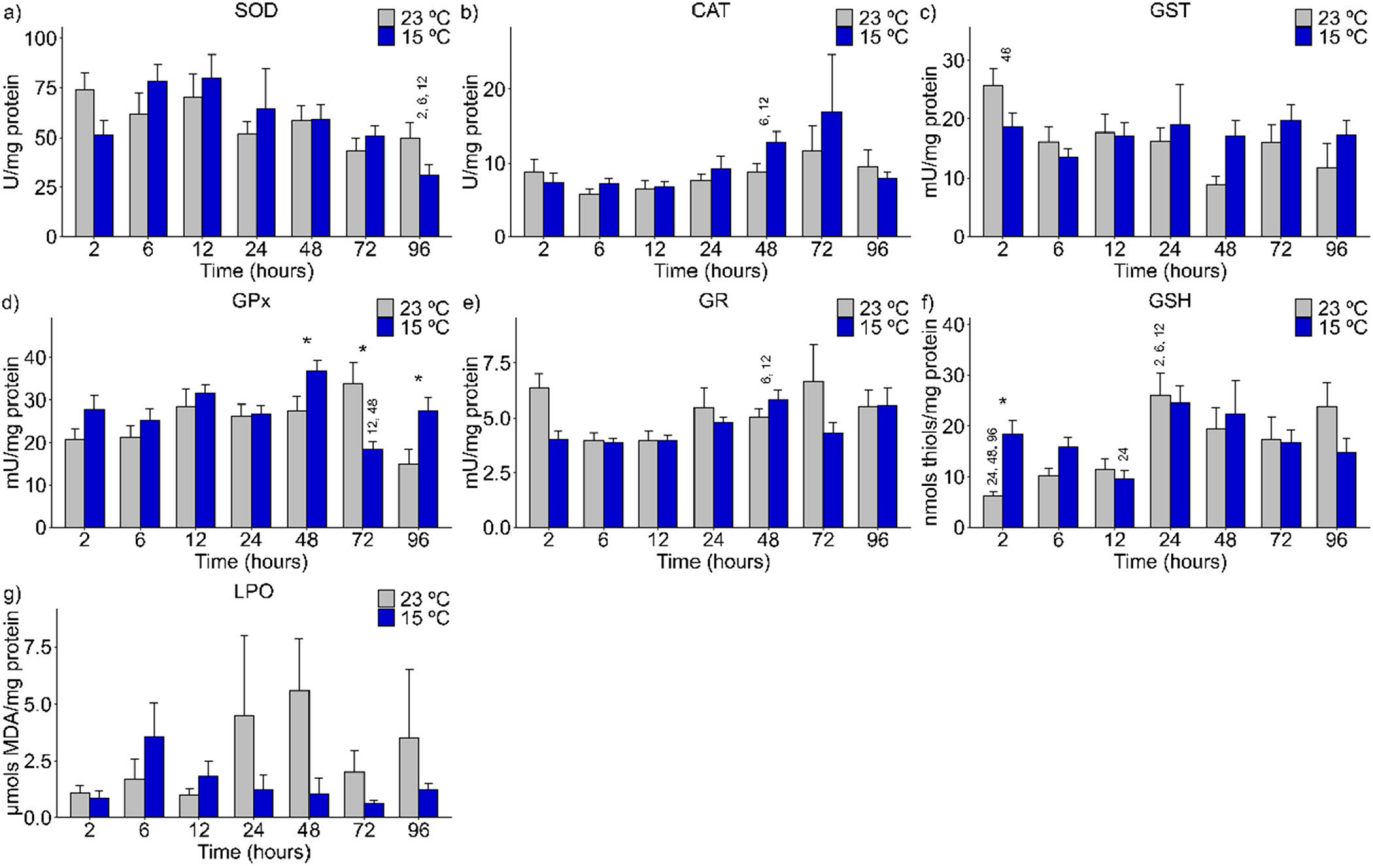
Levels of antioxidant defense biomarkers in the heart of *Astyanax lacustris* exposed to low‐temperature thermal shock (15°C) and their respective controls (23°C). The asterisks (*) represent significant differences (*p* < 0.05) between the control and experimental groups. The numbers above the bars represent the variation between the different exposure times. The values are presented as the means and standard errors of the means. A—SOD, superoxide dismutase; B—CAT, Catalase; C—GST, Glutathione S‐transferase; D—GPx, Glutathione peroxidase; E—GR, Glutathione reductase; F—GSH‐ Reduced glutathione; G—LPO, Lipid peroxidation.

At low temperature (15°C), changes in the GPx (*p* = 0.005) and GSH (*p* = 0.013) markers were observed in the heart. GPx (Figure 7d) activity increased at 48 and 96 h and decreased at 72 h, whereas GSH (Figure 7f) contents increased at 2 h.

The markers SOD (Figure 7a), CAT (Figure 7b), GST (Figure 7c), GR (Figure 7e), and LPO (Figure 7g) did not differ at 15°C compared with the respective 23°C controls in *A. lacustris* heart.

### 3.5 Responses of Oxidative Stress Biomarkers at the Different Temperatures in the Heart of *A. lacustris*

**Figure 8:**
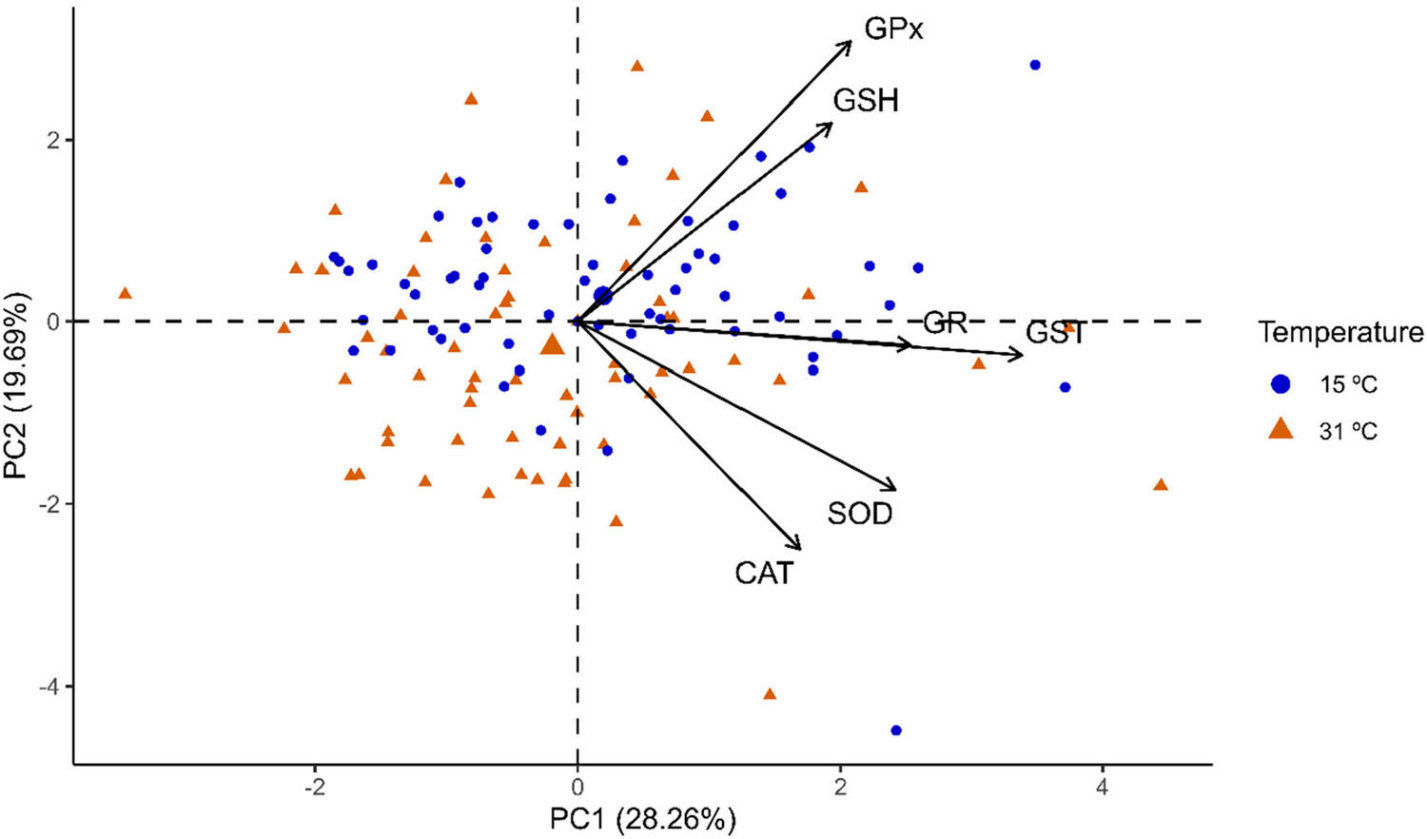
Graph of the principal component analysis (PCA) of biomarkers from the heart of Astyanax lacustris exposed to temperatures of 15°C and 31°C. CAT, catalase; GST, glutathione S‐transferase; GPx, glutathione peroxidase; GR, glutathione reductase; GSH, reduced glutathione; LPO, lipoperoxidation; SOD, superoxide dismutase.

The variation in heart antioxidant defense markers at temperatures of 31°C and 15°C was evaluated using PCA; 47.95% of the variation was explained, with 28.26% explained by the first axis and 19.69% explained by the second axis (Figure 8). The biplot in Figure 8 indicates that the biomarkers SOD, CAT, GST, GPx, GR, GSH, and LPO presented the same correlation at both temperatures (31°C and 15°C).

### 3.6 Integrated Index of Biomarker Response (IBR) for the High (31°C) and Low (15°C) Temperature Treatments in the Heart of *A. lacustris*

**Figure 9:**
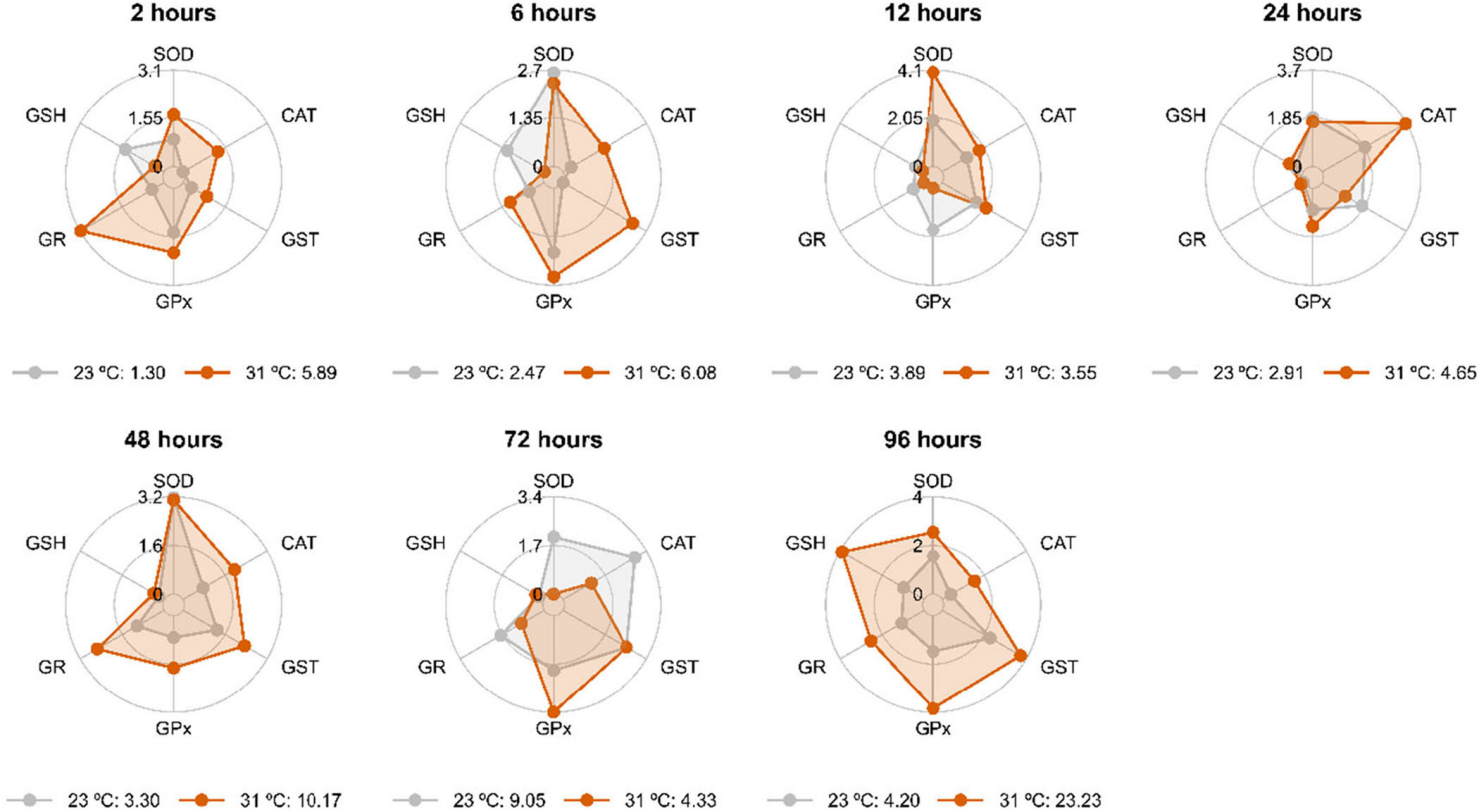
Values of the integrated biomarker response (IBR) index of the heart of Astyanax lacustris exposed to high temperature (31°C) for 2, 6, 12, 24, 48, 72, and 96 h. CAT, catalase; GST, glutathione S‐transferase; GPx, glutathione peroxidase; GR, glutathione reductase; GSH, reduced glutathione; SOD, superoxide dismutase.

**Figure 10:**
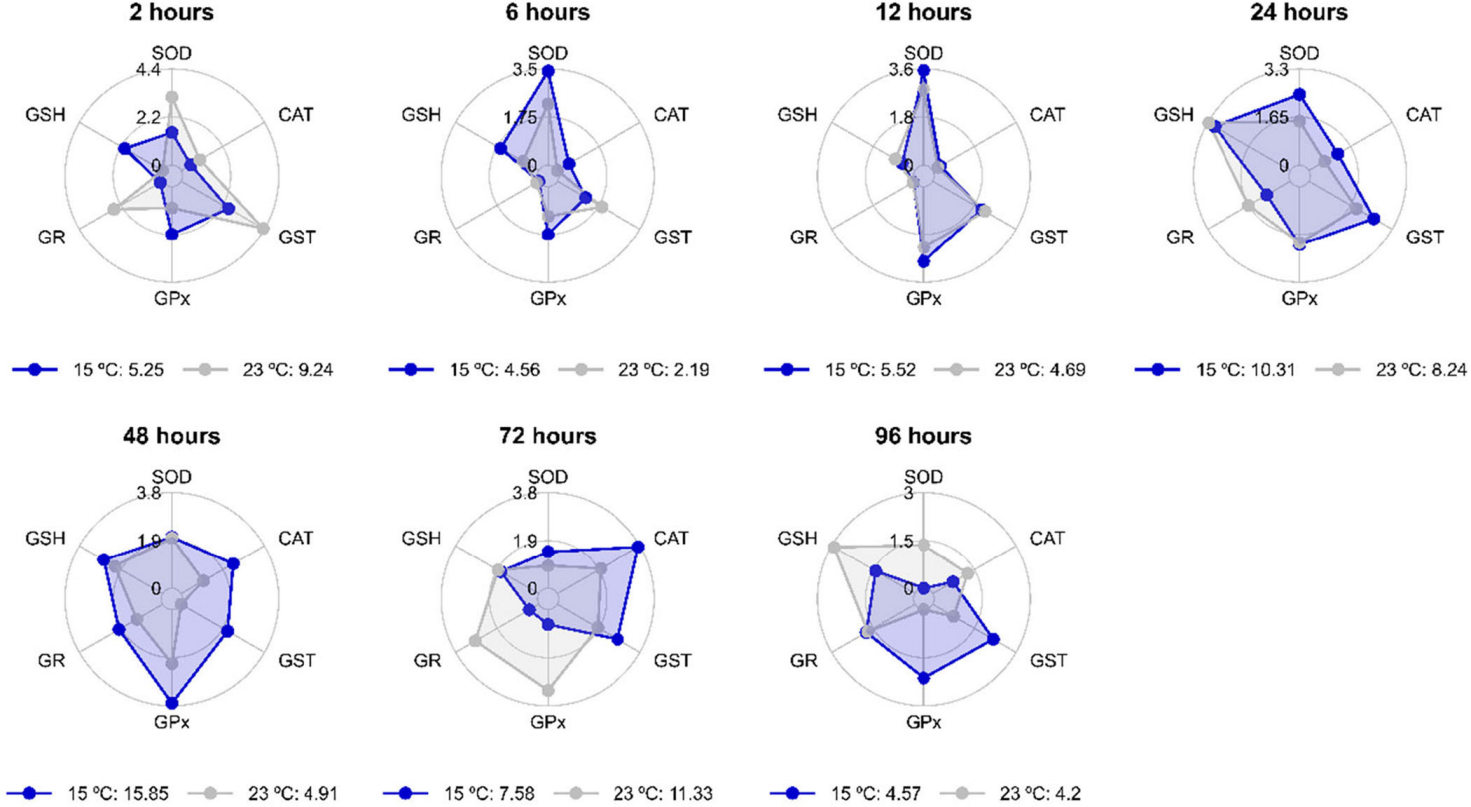
Values of the integrated biomarker response (IBR) index of the heart of *Astyanax lacustris* exposed to low temperature (15°C) for 2, 6, 12, 24, 48, 72, and 96 h. CAT, catalase; GST, glutathione S‐transferase, GPx, glutathione peroxidase; GR, glutathione reductase; GSH, reduced glutathione; SOD, superoxide dismutase.

**Figure 11:**
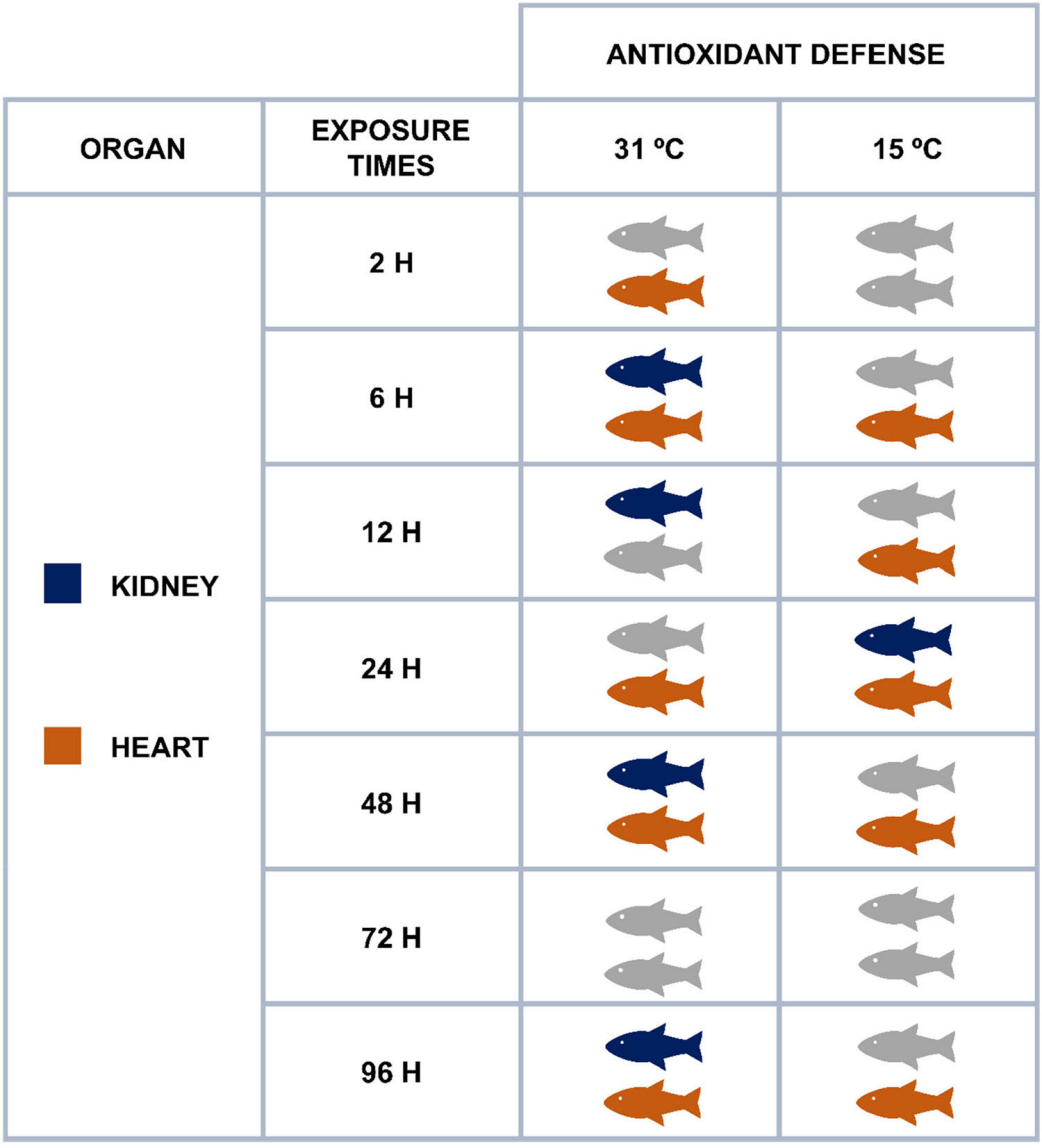
Scheme demonstrating the mobilization of the antioxidant defense system of *Astyanax lacustris* against thermal stress at 31°C and 15°C.

In the heart, the most responsive biomarkers of antioxidant defense after exposure to 31°C at the time points evaluated were as follows: SOD, CAT, GST, GPx, and GR at 2 h; CAT, GST, GPx, and GR at 6 and 48 h; SOD, CAT, and GST at 12 h; CAT and GPx at 24 h; GPx at 72 h; and all antioxidant defense markers (SOD, CAT, GST, GPx, GR, and GSH) at 96 h (Figure 9). After exposure to 15°C, the following were the most responsive biomarkers: GPx and GSH at 2 h; SOD, CAT, GPx, and GSH at 6 h; SOD and GPx at 12 h; SOD, CAT, and GST at 24 and 72 h; CAT, GST, GPx, GR, and GSH at 48 h; and GST and GPx at 96 h (Figure 10). The IBR values were summarized in Figure 11.

### 3.7 Sensitivity Values at 15°C and 31°C

Table 1 shows the rates of variation in the IBR at 31°C and 15°C. The results revealed greater sensitivity of the antioxidant defense system to increased temperature (31°C) in the kidneys at 6, 12, and 96 h and in the heart at 2, 6, 24, 48, and 96 h of exposure. Under reduced temperature (15°C), the highest reactivity occurred at 72 h in the kidneys and at 6 and 48 h in the heart.

## 4 Discussion

In this study, the mortality rate observed during the bioassays was 3.9%, which is considered low for experimental conditions [5]. The deaths were most likely due to individual physiological factors, as the abiotic factors intrinsic to the experiment (dissolved O_2_, ammonia, and pH) were evaluated throughout the experiment. In addition, with increasing exposure time, some significant variations in the analyzed markers were observed in the fish in the control group, and the circadian cycle may have influenced such changes [66]. The circadian system in organisms is responsible for the temporal organization of physiological processes, such as the hormonal regulation of melatonin and cortisol [67]. In addition to being involved in sleep and wakefulness processes, melatonin is capable of neutralizing free radicals and increasing the activity of antioxidant enzymes such as GPx, SOD, and CAT [68, 69].

### 4.1 Effects of High (31°C) and Low (15°C) Temperature Thermal Shock on the Heart and Kidneys of *A. lacustris*

The results of this study for the temperatures tested (15°C and 31°C) showed that thermal stress led to changes in the antioxidant defense system of both tested organs of *A. lacustris*.

SOD and CAT are essential enzymes in the neutralization of ROS, and their responses differed between the organs analyzed. In the kidneys of *A. lacustris* exposed to 31°C, CAT activity increased at 6 and 12 h, coinciding with higher levels of lipid peroxidation at 12 h, and later decreased at 72 h, indicating progression of oxidative stress. In the heart, however, CAT activity did not change, and only SOD activity increased after 12 h at 31°C. Similar patterns have been described in the hearts of Antarctic notothenioids ([30]), in the subtropical species *Rhamdia voulezi* [57], and in tropical fishes [70]. These results suggest that other antioxidant defense systems, such as the Trx/Prx pathway [71] or thiol oxidation [72], may contribute to peroxide detoxification in the heart. Moreover, GST activity increased at 12 h and remained high at 48 and 96 h, while GPx activity decreased at 12 h, reinforcing the susceptibility of cardiac cells to peroxide‐induced damage.

However, for efficient functioning under stress conditions, GST requires glutathione. The demand for glutathione is predominantly met by GR because the reduction of glutathione by GR is less energetically expensive than de novo synthesis [73]. The cytosolic levels of this nonprotein thiol are dependent on its synthesis or on the reduction of GSSG by GR. To maintain homeostasis, GSH levels are usually higher than GSSG levels [74]. In the heart of *A. lacustris*, a delayed response (96 h) of increases in GPx activity and GSH levels was observed, which indicates that thermal stress at 31°C promotes an increased peroxide production response.

Antioxidant enzymes, such as SOD, CAT, and GPx, are recognized as inducible enzymes in fish, and their activities normally increase in response to mild oxidative stress, acting as a compensatory response [75, 76]. These responses, together with the decrease in SOD activity at 31°C and the absence of lipid peroxidation in the heart at both temperatures, may demonstrate that the basal components of the antioxidant defense system in the cardiac tissue of *A. lacustris* are efficient in eliminating hydrogen peroxide during exposure to 31°C, thus preventing damage to cellular structures. In addition, Schleger et al. [20, 24] reported no changes in lipid peroxidation in the liver or gills (31°C) of *A. lacustris* exposed to thermal stress. However, the same was not observed in the kidneys, which presented altered levels of lipid peroxidation. The behavior of oxidative stress markers observed in the heart of *A. lacustris*, compared with other organs of the same species [20, 23, 24, 25] and the kidneys, may demonstrate a greater antioxidant capacity of the heart since the impact of thermal stress on the heart is not limited to the increasing demand for oxygen; it also has significant implications for the functioning of the heart, which is considered the most susceptible organ to temperature variations [30]. Antioxidant responses are related to modulations of heat shock proteins (HSPs) [77]. HSPs act as sensors of redox changes in cells and activate enzymes such as SOD, CAT, and other peroxidases, in addition to stimulating processes that maintain the integrity of mitochondrial membranes (D [78]). Even the constitutive levels of HSP70 increase in advance in tissues in preparation for thermal stress [79]. At high temperatures (31°C), the IBR values of the heart of *A. lacustris* revealed greater mobilization of enzymes of the antioxidant system at 2, 48, and 96 h, and LPO and GSH were positively correlated at this temperature.

Another point to be considered when analyzing the set of responses (SOD, CAT, GPX, and LPO), especially in relation to the possible susceptibility of the heart and kidneys at 31°C for 12 h, is that several researchers suggest that peroxide lipids may act as signaling molecules that initiate the responses of HSPs and other cellular mechanisms to adjust the extent of cellular thermosensitivity (C [80]; C [81]). This mechanism is possible when oxidative stress remains below the threshold level to cause cellular damage or when the animal is more resistant to lipid peroxidation [82]. In such a case, an acclimation process is induced to achieve physiological homeostasis (C [81]).

As a classical response to thermal stress, the antioxidant defenses of fish organs and tissues are expected to increase (D [41, 78]). However, species that occupy different thermal niches exhibit distinct peaks of biomarkers throughout the acclimation process (D [75]). Under natural conditions, the water temperature of the Iguaçu River varies between 15.5°C and 28.8°C [33]. In aquatic environments, a decrease in water temperature is linked to rapid variations in solar heat, the renewal of water in ponds and reservoirs, seasonal changes, and climatic events. These low temperatures have the potential to cause stress and physiologically compromise organisms [83, 84]. However, in the present study, both the heart and kidney of *A. lacustris* were tolerant to low‐temperature thermal stress (15°C) after up to 72 h of exposure, as evidenced by a reduction in ROS levels in the kidneys. This suggests that up to 72 h of exposure to cold may have led to a reduction in cellular metabolic activity, resulting in lower oxygen use and a consequent reduction in ROS production. In general, the evaluated tissues presented a lower and a slower response to cold thermal stress (15°C) than to thermal stress (31°C), as evidenced by the increase in GR activity and ROS production in the kidneys and GPx activity in the heart after 96 h of exposure.

In addition, GPx activity and the GSH concentration did not change during the rinses, which may be related to preferential pathways for the activation of antioxidant mechanisms, since the kidneys exhibit glutathione‐dependent mobilization and potentially present have higher levels of these biomarkers precisely because it is part of the first stress signaling pathway (Z [9]). The responses of the heart and kidneys to the markers evaluated corroborate the findings of several studies; specifically, the responses of the antioxidant defense system to thermal stress are specific to the organ, species, temperature, and duration of exposure.

The IBR results for the heart of *A. lacustris* at 31°C corroborate the sensitivity data for that same temperature. The IBR results revealed greater mobilization of the antioxidant system at 96 h of exposure, which was also the time that presented the highest degree of sensitivity, and the same phenomenon was observed at 2, 6, and 48 h of exposure. A greater mobilization of glutathione‐dependent biomarkers was also observed at 96 h of exposure to 31°C, which may indicate that, during prolonged periods of stress, glutathione may be a key molecule that modulates the stress response, as may the denaturation of enzymes [85]. The kidneys showed greater sensitivity at 12 and 96 h of exposure to 31°C. The IBR values revealed greater mobilization of antioxidant enzymes at 96 h, with activation of GSH and GR followed by the mobilization of GPx and SOD 12 h later.

The same pattern of mobilization of the antioxidant defense system was observed in the IBR results for heart sensitivity at 15°C. Although the antioxidant mechanisms were activated to a lesser degree than at warmer temperature (31°C), at 48 h of exposure, the heart showed greater sensitivity, and the IBR values indicate greater mobilization of biomarkers at the same exposure times. Under cold stress (15°C), the exposure time that presented the greatest sensitivity and greatest mobilization of biomarkers in the kidney tissue was 72 h. This correlation between the IBR values and the responsiveness of the biomarkers was also reported by Madeira et al. [40], who observed that the most responsive exposure time (with a significant increase in biomarker levels) was 7 days under high temperature. This finding was supported by the results of the IBR index, which showed higher values under high temperature relative to the control group [40].

The results of the kidney PCA analysis indicated that glutathione‐dependent biomarkers were more strongly associated with high temperature (31°C), whereas first‐line biomarkers such as CAT and SOD were more strongly associated with low temperature (15°C). This finding is in agreement with the IBR results, which also demonstrate the tendency of the mobilization of glutathione‐dependent biomarkers at high temperature, as observed in the mobilization of GSH and GR at 96 h and GPx at 12 h of exposure, as well as at low temperature with the mobilization of CAT at 72 h of exposure. The PCA of the heart of *A. lacustris* indicated no variation in the mobilization of biomarkers between the different temperatures tested.

## 5 Conclusions

The response of the antioxidant defense system of the heart and kidneys of *A. lacustris* exposed to high (31°C) and low (15°C) temperature shock was evaluated. In general, the evaluated tissues presented a lower and slower thermal response to cold stress (15°C) than to heat stress (31°C).

Both tissues showed greater sensitivity when exposed to higher temperatures, and these results are corroborated by the IBR values, which also revealed greater mobilization of biomarkers at high temperatures. Through PCA of the kidneys, we observed a trend toward associations between glutathione‐dependent biomarkers at high temperature and first‐line defense biomarkers, such as SOD and CAT, at low temperature, whereas PCA of the heart indicated that the biomarkers were associated equally with both temperatures tested. In general, high‐temperature thermal stress seems to cause greater impacts and demand greater activation of the antioxidant system than low‐temperature thermal shock for the experimental times and conditions tested here.

## Author Contributions

**Ana Paula Nascimento Corrêa:** conceptualization, formal analysis, data curation, investigation, writing – original draft preparation. **Luiz Neves Neto:** data curation, investigation, writing – original draft preparation. **Maria Rosa Dmengeon Pedreiro de Souza:** conceptualization, methodology, writing – review and editing. **Niumaique Gonçalves da Silva:** data curation, formal analysis, investigation. **Jonathan Ratko:** data curation, formal analysis, investigation. **Ananda Karla Alves Neundorf:** data curation. **Ieda Cristina Schleger:** conceptualization and investigation. **Tatiana Herrerias:** writing – review and editing. **Lucélia Donatti:** conceptualization, methodology, investigation, resources, data curation, writing – review and editing, supervision, project administration, funding acquisition.

## Conflicts of Interest

The authors declare no conflicts of interest.

## Supporting information


**TABLE 1:** ANOVA Results for the Antioxidant Defense EnzymES in the Heart of *Astyanax lacustris*. **TABLE 2:** ANOVA RESULTS FOR THE GSH CONTENTS AND LPO OF THE HEART OF *Astyanax lacustris*. **TABLE 3:** ANOVA RESULTS OF THE DEFENSE ANTIOXIDANT ENZYMES OF THE MID‐POSTERIOR KIDNEY OF *Astyanax lacustris*. **TABLE 4:** ANOVA RESULTS FOR OXIDATIVE DAMAGE, GSH, AND ROS IN THE BRAIN OF *Astyanax lacustris*.

## Data Availability

The data that support the findings of this study are available from the corresponding author upon reasonable request.
