# Supplementary material for: Antioxidant Defenses in the Kidneys and Heart of the Freshwater Fish Astyanax lacustris Subjected to High (31°C) and Low (15°C) Temperatures
Source: Cell Biochem Funct. 2025 Oct 28;43(11):e70133. doi: 10.1002/cbf.70133 (PMC12560206; doi:10.1002/cbf.70133)
Supplement: Supplementary file 1 — TABLE 1: ANOVA Results for the Antioxidant Defense EnzymES in the Heart of Astyanax lacustris. TABLE 2: ANOVA RESULTS FOR THE GSH CONTENTS AND LPO OF THE HEART OF Astyanax lacustris. TABLE 3: ANOVA RESULTS OF THE DEFENSE ANTIOXIDANT ENZYMES OF THE MID‐POSTERIOR KIDNEY OF Astyanax lacustris. TABLE 4: ANOVA RESULTS FOR OXIDATIVE DAMAGE, GSH, AND ROS IN THE BRAIN OF Astyanax lacustris. [file CBF-43-e70133-s001.docx]

**SUPPLEMENTARY MATERIAL**

Tables of the F and p values of the biomarkers measured in the kidneys and heart of *Astyanax lacustris*

TABLE 1 - ANOVA RESULTS FOR THE ANTIOXIDANT DEFENSE ENZYMES IN THE HEART OF *Astyanax lacustris*

| **ANOVA statistics** | | | **SOD** | **CAT** | **GST** | **GPx** | **GR** |
| --- | --- | --- | --- | --- | --- | --- | --- |
| **Temperature**  **X**  **Time** | 31 °C | **F** | 2.235 | 1.254 | 1.739 | 3.777 | 1.112 |
|  |  | ***p*** | 0.045* | 0.285 | 0.118 | 0.002* | 0.360 |
|  | 15 °C | **F** | 1.656 | 0.672 | 1.817 | 3.347 | 1.931 |
|  |  | ***p*** | 0.140 | 0.672 | 0.103 | 0.005* | 0.083 |
| **Time** | 31 °C | **F** | 2.635 | 1.766 | 4.450 | 4.447 | 2.235 |
|  |  | ***p*** | 0.020* | 0.112 | < 0.001* | < 0.001* | 0.045* |
|  | 15 °C | **F** | 4.327 | 2.958 | 2.225 | 2.918 | 3.655 |
|  |  | ***p*** | < 0.001* | 0.011* | 0.047* | 0.011* | 0.002* |

NOTE: SOD, superoxide dismutase; CAT, catalase; GST, glutathione S-transferase; GPx, glutathione peroxidase; GR, glutathione reductase. Asterisks indicate significant differences (p < 0.05).

TABLE 2 - ANOVA RESULTS FOR THE GSH CONTENTS AND LPO OF THE HEART OF *Astyanax lacustris*

| **ANOVA statistics** | | | **GSH** | **LPO** |
| --- | --- | --- | --- | --- |
| **Temperature**  **X**  **Time** | 31 °C | **F** | 3.242 | 0.530 |
|  |  | ***p*** | 0.006* | 0.784 |
|  | 15 °C | **F** | 2.868 | 2.159 |
|  |  | ***p*** | 0.013* | 0.054 |
| **Time** | 31 °C | **F** | 2.110 | 3.682 |
|  |  | ***p*** | 0.059 | 0.003* |
|  | 15 °C | **F** | 4.865 | 1.466 |
|  |  | ***p*** | < 0.001* | 0.198 |

NOTE: GSH, reduced glutathione; LPO, lipoperoxidation. Asterisks indicate significant differences (p < 0.05).

TABLE 3 - ANOVA RESULTS OF THE DEFENSE ANTIOXIDANT ENZYMES OF THE MID-POSTERIOR KIDNEY OF *Astyanax lacustris*

| **ANOVA statistics** | | | **SOD** | **CAT** | **GST** | **GPx** | **GR** | **G6PDH** |
| --- | --- | --- | --- | --- | --- | --- | --- | --- |
| **Temperature**  **X**  **Time** | 31 °C | **F** | 2.163 | 3.147 | 2.532 | 1.383 | 2.387 | 0.965 |
|  |  | ***p*** | 0.615 | 0.010* | 0.032* | 0.239 | 0.215 | 0.458 |
|  | 15 °C | **F** | 2.240 | 1.389 | 1.050 | 0.470 | 2.929 | 0.433 |
|  |  | ***p*** | 0.054 | 0.241 | 0.406 | 0.828 | 0.016* | 0.853 |
| **Time** | 31 °C | **F** | 8.019 | 1.521 | 5.360 | 4.082 | 7.913 | 1.652 |
|  |  | ***p*** | < 0.001* | 0.190 | < 0.001* | 0.002* | < 0.001* | 0.151 |
|  | 15 °C | **F** | 6.281 | 1.850 | 0.595 | 2.216 | 8.952 | 0.867 |
|  |  | ***p*** | < 0.001* | 0.111 | 0.733 | 0.057 | < 0.001* | 0.526 |

NOTE: SOD, superoxide dismutase; CAT, catalase; GST, glutathione S-transferase; GPx, glutathione peroxidase; GR, glutathione reductase; G6PDH, glucose-6-phosphate dehydrogenase. Asterisks indicate significant differences (p < 0.05).

TABLE 4 - ANOVA RESULTS FOR OXIDATIVE DAMAGE, GSH, AND ROS IN THE BRAIN OF *Astyanax lacustris*

| **ANOVA statistics** | | | **GSH** | **LPO** | **PCO** | **ROS** |
| --- | --- | --- | --- | --- | --- | --- |
| **Temperature**  **X**  **Time** | 31 °C | **F** | 1.696 | 2.626 | 1.746 | 0.740 |
|  |  | ***p*** | 0.141 | 0.027* | 0.128 | 0.620 |
|  | 15 °C | **F** | 0.510 | 0.941 | 0.976 | 3.315 |
|  |  | ***p*** | 0.798 | 0.474 | 0.452 | < 0.001* |
| **Time** | 31 °C | **F** | 1.353 | 5.045 | 4.817 | 12.637 |
|  |  | ***p*** | 0.251 | < 0.001* | < 0.001* | < 0.001* |
|  | 15 °C | **F** | 1.845 | 0.486 | 1.635 | 5.352 |
|  |  | ***p*** | 0.109 | 0.816 | 0.157 | < 0.001* |

NOTE: GSH, reduced glutathione; LPO, lipoperoxidation; PCO, protein carbonylation; ROS, reactive oxygen species. Asterisks indicate significant differences (p < 0.05).
